# Evaluating the capacity of species distribution modeling to predict the geographic distribution of the mangrove community in Mexico

**DOI:** 10.1371/journal.pone.0237701

**Published:** 2020-08-20

**Authors:** Karla Rodríguez-Medina, Carlos Yañez-Arenas, A. Townsend Peterson, Jorge Euán Ávila, Jorge Herrera-Silveira

**Affiliations:** 1 Departamento de Recursos del Mar, CINVESTAV-IPN, Unidad Mérida, Mérida, Yucatán, Mexico; 2 Laboratorio de Ecología Geográfica, Unidad de Biología de la Conservación, Parque Científico y Tecnológico de Yucatán, Unidad Académica Sisal—Facultad de Ciencias, UNAM, Mérida, Yucatán, Mexico; 3 Biodiversity Institute, University of Kansas, Lawrence, KS, United States of America; Swedish University of Agricultural Sciences, SWEDEN

## Abstract

Mangroves are highly productive ecosystems that provide important environmental services, but have been impacted massively in recent years by human activities. Studies of mangroves have focused on their ecology and function at local or landscape scales, but little has been done to understand their broader distributional patterns or the environmental factors that determine those distributions. Species distribution models (SDMs), have been used to estimate potential distributions of hundreds of species, yet no SDM studies to date have assessed mangrove community distributions in Mexico (the country with the fourth largest extent of this ecosystem). We used maximum entropy approaches to model environmental suitability for mangrove species distributions in the country, and to identify the environmental factors most important in determining those distributions. We also evaluated whether this modeling approach is adequate to estimate mangrove distribution as a community across Mexico. Best models were selected based on statistical significance (AUC ratio), predictive performance (omission error of 5%), and model complexity (Akaike criterion); after this evaluation, only one model per species met the three evaluation criteria. Environmental variable sets that included distance to coast yielded significantly better models; variables with strongest contributions included elevation, temperature of the coldest month, and organic carbon content of soil. Based on our results, we conclude that SDMs can be used to map mangrove communities in Mexico, but that results can be improved at local scales with inclusion of local variables (salinity, hydroperiod and microtopography), field validations, and remote sensing data.

## Introduction

Mangroves are highly productive ecosystems that provide important ecosystem services [1–3]. Given their great ecological and economic relevance, these ecosystems have been studied widely: local studies of species composition [4–9], studies of primary productivity and carbon sequestration [10–14], population ecology [15–17], etc. Most of these studies are carried out at local scales, generating information crucial to understanding the ecology and function of mangroves. However, is difficult to generalize or quantify relevant aspects for conservation of these ecosystems, such as the regional distributions of species, factors that limit their ranges, and loss of surface area, since large areas of mangroves are lost yearly in many regions as a consequence of human impacts [18, 19]. Global mangrove forest conservation requires ample support given that few mangrove areas have increased or recovered thanks to well-implemented public policies [20].

Several remote sensing methods have been employed in recent decades to study spatial mangroves at different spatial scales. At regional extents, satellite images are used to classify and map mangrove communities in the present, and the loss of these communities over time [21, 22]. Other aspects that can be assessed via remote sensing methods include evaluations of the health and condition of the community, reforestation success, and productivity [21]. Remote sensing data products also provide crucial data inputs for species distribution models (SDMs), commonly used techniques to map species’ geographic ranges and generate spatial estimates of environmental suitability [23], in the form of precise digital elevation models, spatial summaries of vegetation characteristics, etc.

Species distribution models (SDM) have been used to estimate potential geographic distributions of hundreds of species and plant communities [24–26]. Nonetheless, only one study to date has used these tools to model distributions of mangrove species: present and future distributions of various mangrove species were estimated to understand potential effects of climate change and sea level rise [27]. To date, no studies have evaluated the performance of SDM tools in mapping and estimating mangrove species’ distributions at finer scales, which would be important, particularly in countries with high richness and coverage of mangrove communities. Modeling on this scale would allow development of maps at higher spatial resolution and detail across the area than currently exist.

Six of the 70 mangrove species are found in Mexico, which has the fourth most extensive mangrove systems of any country in the world [3, 22, 23]. Still, human activities such as tourism, urbanization, agriculture, livestock, and aquaculture development, have impacted mangrove ecosystems in this country [3, 28–30]. Moreover, global changes, such as sea-level rise and temperature increase, are anticipated to decrease the extent of mangrove communities in the future, particularly along the coasts of Mexico, Central America, and the Caribbean [31].

Aware of this predicament, the Mexican government, via the National Commission for the Knowledge and Use of Biodiversity (CONABIO, in Spanish), invested 43,816,000 Mexican pesos (almost US$2M) over more than a decade in a mangrove monitoring program through satellite images, and verification with helicopter-acquired photographs [3]. Thanks to this program, Mexico has uniquely detailed maps of the geographic distribution of its mangroves, at least as a community. These maps offer a snapshot in time, yet mangrove communities are dynamic, given human impacts and environmental changes including climatic fluctuations, sea level rise, accretion, erosion, etc. [32–34]. Improvement of this view requires detailed information about species composition, and understanding environmental requirements associated with the distribution of each mangrove species.

SDMs offer a tool by which to understand spatial changes within mangrove communities, their composition and relationships to environmental attributes at broad spatial scales (i.e. country level, Mexico). However, to that end, it is necessary to evaluate the performance of these models and determine whether environmental predictors of the distribution exist and can be identified for each species. This study therefore had the following objectives: (1) generate and select best SDMs for each species, (2) identify environmental variables of greatest importance in explaining mangrove species distributions in the country, and (3) map mangrove communities of Mexico as an aggregate of environmental suitability for each species individually, and evaluate whether these models are close to current mangrove distributions in Mexico. These models were developed for each species separately, and in terms of the community as a whole.

## Materials and methods

### 1) Input data

Occurrence data were obtained for the four most common and representative mangrove species in Mexico: *Rhizophora mangle* L., *Laguncularia racemosa* (L.) C.F. Gaertn., *Avicennia germinans* (L.) L., *Conocarpus erectus* L. The other two species, *Avicennia bicolor* and *Rhizophora harrisoni*, were not considered, since they have restricted distributions in the country with only a few isolated populations in the states of Chiapas and Oaxaca [35, 36]. Records were extracted from the scientific literature and from the GBIF digital biodiversity data portal (https://www.gbif.org/). For each species, records were assessed to eliminate data from outside of Mexico, data that were clearly erroneous (i.e. outside coastal areas, records falling in the sea, etc.), and duplicated occurrence records.

Afterwards, based on a digital elevation model (www.inegi.org.mx/app/geo2/elevacionesmex/) and a data layer summarizing distributions of rivers of Mexico (www.inegi.org.mx/temas/hidrografia/default.html#Descargas), we eliminated records that appeared to be inaccurate.

We performed an environmental outlier detection analysis based on values of climate, soil, and vegetation: we used the ‘quikclean’ function modified from the ‘biogeo’ package [37] in R [38], which searches for records falling 1.5-fold beyond the interquartile range of data [37]. In addition, a temporal filtering was realized, eliminating historical records (before 2000) that did not coincide temporally with some of the environmental variables, and could represent populations that no longer exist. Finally, we split our final databases into two equal groups for model and evaluation using the checkerboard method of the ENMEval package [39] in R [38].

Defining a geographic area for analysis with a biological basis avoids many problems in interpretation; for this reason, an accessible area was defined for the four mangrove species [39, 40] that represents a hypothesis of historical accessibility for each species [41]. The dispersal features of each species as well as geographic barriers were taken into account for the delineation of this area. Therefore, considering broad latitudinal distributions and even-broader dispersal ‘reach,’ we examined the entire coastline of the country and generated a polygon of 35 km inland from the coast (using buffering routines in ArcMAP 10.2) based on the assumption that all mangrove communities are found <30 km from the coast [42].

We selected 31 environmental layers for initial development of models (Table 1) that are potentially important in delimiting mangrove communities at regional scales [43, 44]. Procedures by which these layers were obtained and processed are as follows.

**Table 1:** All environmental predictors used in the models.

Variable	*Avicennia germinans*		*Conocarpus erectus*		*Laguncularia racemosa*		*Rhizophora mangle*	
	PC	PI	PC	PI	PC	PI	PC	PI
Annual mean temperature	0.3	0	1	0.3	0.4	0	0.9	0.7
Mean diurnal range	0.2	1.2	0.2	0.3	0.4	2.9	0.2	0.9
Isothermality	2.3	1	0.1	0.3	0	0	0.3	0.4
Temperature seasonality	0.8	0.1	0.4	0	0	0	0	0.1
Maximum temperature of warmest month	0.1	0	0	0	0	0	0	0.5
Minimum temperature of coldest month	3.7	12.9	7.9	13.9	5	2	4	8.3
Annual temperature range	1	1.2	0.5	0.1	0.7	0.2	0.9	0.3
Mean temperature of warmest quarter	2	4.3	0	0	0.3	1.8	0.4	2
Mean temperature of coldest quarter	1.7	1.6	3.6	0.2	1.4	0.9	0.6	0
Annual precipitation	1.1	4.9	0	0	0.5	8	0.3	0.6
Precipitation of wettest month	0.4	0.9	0	0	0	0	0.7	1.8
Precipitation of driest month	12.5	4.7	0.1	1.3	14.9	0	0.1	0.7
Precipitation seasonality	1	0.9	0	0.5	0.3	2.3	1.4	6.8
Precipitation of wettest quarter	0.5	2.1	0.2	4.2	0.5	1	0	0
Precipitation of driest quarter	12.7	3.8	15.3	3.6	0	0	13.3	3.1
Depth to bedrock	…	…	5	4.8	4.8	5.8	6.3	6.3
Bulk density	…	…	0.2	0	1.6	2.7	0.7	2.2
Cation-exchange capacity	…	…	0.1	0	0	0.6	0.2	0.5
Clay content	…	…	2	3.2	0.1	1.6	1.1	2.6
Coarse fragments	…	…	1.4	6.6	1.3	2.2	0.7	1.1
Soil organic carbon	…	…	12.6	2.6	9.1	6.8	16.6	14.8
pH index	…	…	0.3	4.7	0.6	3.3	0.8	3.3
Silt content	…	…	0	0.1	0	0.6	0.1	0.6
Sand content	…	…	0.9	0	0	0	0.3	0.3
Distance to coast	43.4	44.9	42.9	45.4	44.3	24.8	43.5	37.1
Digital terrain model	12	10.9	0.7	0.5	8.5	6.2	6.5	4.8
Maximum NDVI value	0.3	0.4	…	…	…	…	…	…
Mean NDVI value	1.6	2.5	…	…	…	…	…	…
Minimum NDVI value	1.6	1.2	…	…	…	…	…	…
NDVI range	0.1	0.1	…	…	…	…	…	…
NDVI standard deviation	0.6	0.2	…	…	…	…	…	…

Percentage contribution (PC) and permutation importance (PI) of environmental variables used to model the potential distribution of Mexican mangroves. Variables without data (…) were not used in the final model of that species.

#### Climate layers

These data layers were downloaded from the Neotropical Bioclimate database, summarizing annual tendencies derived from monthly temperature and precipitation data for 1910–2009 in Mexico (http://idrisi.uaemex.mx/distribucion/superficies-climaticas-para-mexico) [45]. Although 19 data layers make up the dataset, we only used 15, since mean temperature of wettest quarter, mean temperature of driest quarter, precipitation of warmest quarter, and precipitation of coldest quarter possess odd and unrealistic artefacts across geography [46].

#### Soil and topography layers

Nine data layers summarizing characteristics of soil were obtained, corresponding to the top 5 cm of soil, from www.soilgrids.org [47]. A digital elevation model was also used; this model was specifically elaborated for Mexico by INEGI (www.inegi.org.mx/app/geo2/elevacionesmex/).

#### Distance to coast

We created a distance-to-coast layer using Euclidean distances, with the Spatial Analyst tool in ArcMAP 10.2. We determined the coastline based on a vector layer of the political limits of Mexico [48].

#### Vegetation layers

Using data from the Moderate Resolution Spectroradiometer (MODIS, https://modis.gsfc.nasa.gov/), layers summarizing normalized difference vegetation index data (NDVI) between 2002 and 2016 were generated, including mean, maximum, minimum, range, and standard deviation.

All layers were used at a spatial resolution of 30” (~1 km^2^) and cropped to the limits of Mexico and of our accessible area hypothesis.

### 2) Model development

The Maxent algorithm 3.4.1 [49] was used to estimate environmental suitability and potential geographic distributions of the four mangrove species. With the KUENM R package [48], a series of candidate Maxent models under different combinations of parameter settings was developed in R 3.5.0 [38]. These combinations included nine regularization multiplier (0.1, 0.5, 1.0, 1.5, 2, 3, 4, 5, 8) values, five feature class combinations (l, lq, lqp, lqpt, lqpth: where ‘l’ = linear, ‘q’ = quadratic, ‘p’ = product, ‘t’ = threshold, and ‘h’ = hinge), and 13 different sets of environmental variables. These sets are as follows: (1) all layers (hereafter, All); (2) only climate layers (Clim); (3) only soil layers (Soil); (4) only vegetation layers (Veg); (5) all layers except climate layers (dvts); (6) all layers except soil layers (dvct); (7) all layers except vegetation layers (dcts); (8) all except topography layers (dvcs); (9) vegetation, climate, and soil (vcs); (10) vegetation, climate, and topography (vct); (11) vegetation, climate, topography and soil (vcts); (12) vegetation, topography and soil (vts); and (13) climate, topography and soil (cts). As such, we developed and evaluated a total of 9 x 5 x 13 = 585 candidate models per species.

Best models were selected based on three criteria, as follows. (1) Statistical significance: we computed the area under the curve ratio (AUC ratio) of the partial receiver operating characteristic curve (pROC). In this test, the AUC ratio deviates from unity as the pROC curve improves with respect to null expectations [50]. We defined an acceptable omission error criterion of 5% and a bootstrap resampling (500 replicates) of 50% of evaluation data. Probabilities were obtained by counting the number of bootstrap replicates with AUC ratios of ≤1 [51]. (2) Predictive performance: among significant models, after thresholding with an acceptable omission error criterion of 5%, we selected those with omission rates (OR) ≤5%. (3) Model complexity: the Akaike information criterion (corrected for small sample size, or AICc) was used to select the models from among those meeting the first two criteria that had the least complexity (i.e. models within 2 AICc units of the minimum value among that population of candidate models) [52, 53].

A final set of models was built using the Maxent bootstrap functionality (80% of training and 20% of evaluation data) with five replicates, the ‘logistic’ output and 10,000 of background points. To convert Maxent suitability models into binary maps of presence or absence of suitable environmental conditions, a threshold of allowable omission (*E* = 5%; this percentage was determined based on our level of confidence in the training data) was established over the median of the five replicates. Plots and graphics were generated in ggplot2 package [54] in R [37]. We used the percentage contribution (PC) and permutation importance (PI) to assess the contribution of each environmental variable to the best model [50, 55, 56, 57].

### 3) Richness and community map

The community map was developed as the sum of binary rasters of the species distribution models in ArcGIS 10.2, following ideas for community-level predictions from SDMs [58]. Our final, deliberately conservative predictions of distribution of mangrove as a community, were defined as the areas in which all mangrove species were predicted by our models as finding suitable conditions.

### 4) Final evaluation

To evaluate how well our modeling efforts predicted the actual distribution of the mangrove community, we used the shapefile of mangrove distribution from CONABIO [3], cropped to the polygon of our calibration area (see above). A binary raster (presence/absence) was developed from these two inputs: the mangrove area from CONABIO was designated with value 1 (presence) and outside this area but within our accessible area hypothesis was designated with value 0 (absence). We cast 10,000 random points on this raster, and each point was assigned the value of the raster. We constructed a confusion matrix from the points, and the following metrics were calculated: true skill statistic (TSS) [59], correct classification rate, omission error, and commission error.

## Results

Initial presence records for the four mangrove species in Mexico were 7860 for *A*. *germinans*, 6071 for *C*. *erectus*, 6857 for *L*. *racemosa*, and 12318 for *R*. *mangle*. However, our cleaning processes involving removing points falling inland and duplicate values left only 188, 203, 172, and 209 records for the four species, respectively.

For each mangrove species only one model met the three criteria (statistical significance, predictive performance, model complexity). In general, selected predictor sets and Maxent settings were particular to each species, with the exception of *A*. *germinans* and *R*. *mangle*, which coincided in features and RM value (Table 2).

**Table 2:** Model parameter settings with best predictive capacity in models selected.

Species	Best setting			AUC ratio	OR	AICc	Number of parameters
	Set	RM	Features				
*Avicennia germinans*	dvct	1.5	lqpth	1.717	0.19	3984	86
*Conocarpus erectus*	dcts	1.5	lq	1.775	0.261	3927	31
*Laguncularia racemosa*	dcts	0.5	L	1.736	0.145	3413	24
*Rhizophora mangle*	dcts	5	lqpth	1.739	0.264	4227	42

RM = regularization multiplier, AUC = area under the curve, OR = omission rate, AICc = Akaike information criterion. Each letter in column “Set” is a class of predictors: d = distance to coast, c = climate variables, s = soil variables, t = topography (digital terrain model), v = vegetation variables.

**Fig 1:**
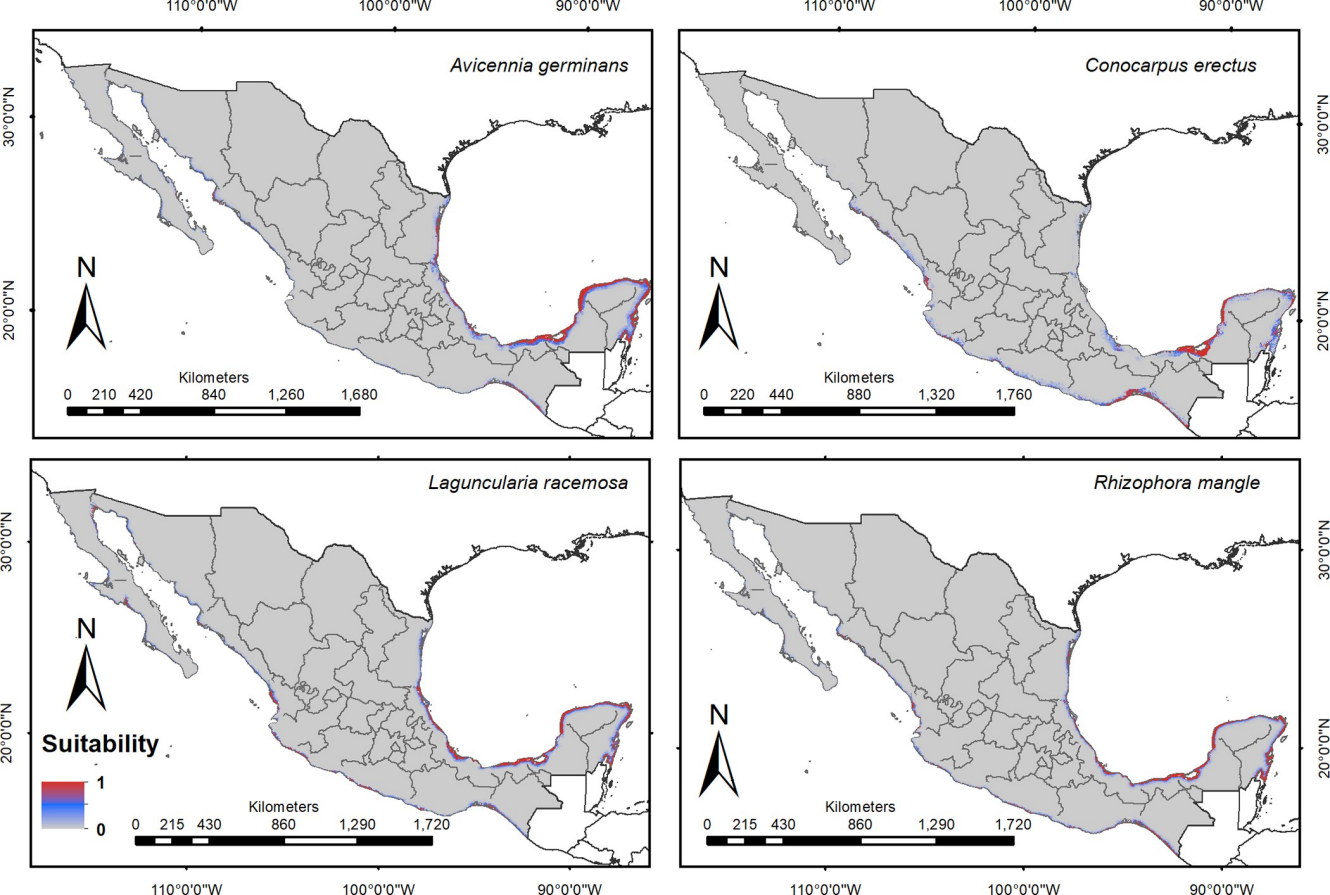
Environmental suitability patterns modeled for the four mangrove species in Mexico. Red indicates highest suitability; blue areas are less suitable; gray areas were excluded from model fitting.

**Fig 2:**
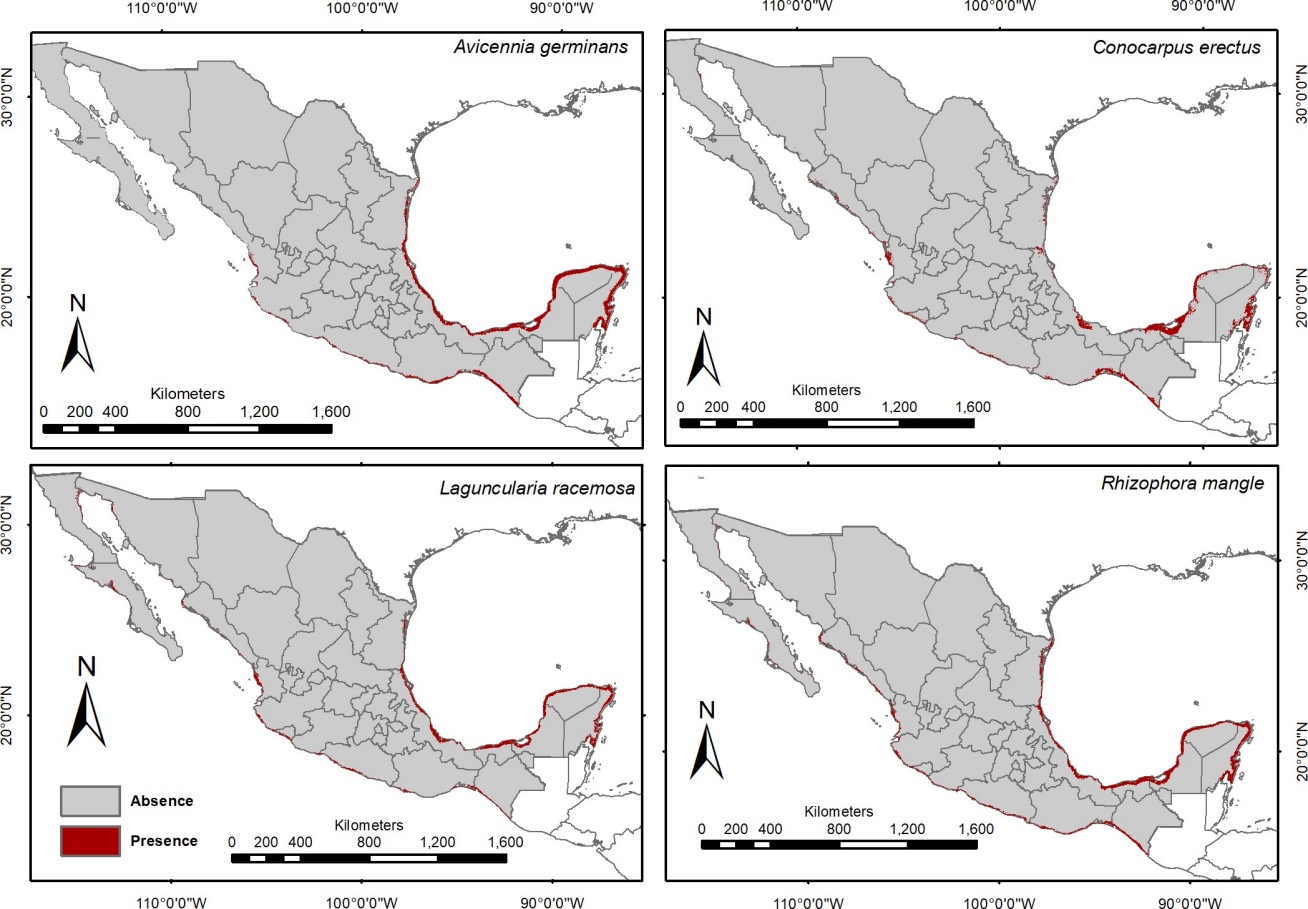
Distribution maps of the four mangrove species, derived from environmental suitability models. Red indicates the potential distribution of mangroves.

Higher environmental suitability was estimated for each of the four species along the Gulf of Mexico compared to the Pacific Coast of western Mexico. *Rhizophora mangle* had the broadest distribution range in the country (50,769 km^2^), followed by *A*. *germinans* (49,882 km^2^), *L*. *racemosa* (36,594 km^2^), and *C*. *erectus* (35,840 km^2^). Suitable areas for *C*. *erectus* were restricted to parts of the Gulf of Mexico coast, and more towards inland areas with freshwater sources (i.e., Vega de Alatorre and Laguna de Alvarado, in Veracruz, parts of Tabasco and Campeche, the northeastern coast of Yucatan, and southern Quintana Roo). The distribution of *R*. *mangle* was broad in the country, yet areas with high environmental suitability were restricted to the Yucatan Peninsula, and to the coasts of Chiapas, Oaxaca, and Jalisco, on the Pacific coast (Figs 1 and 2).

**Fig 3:**
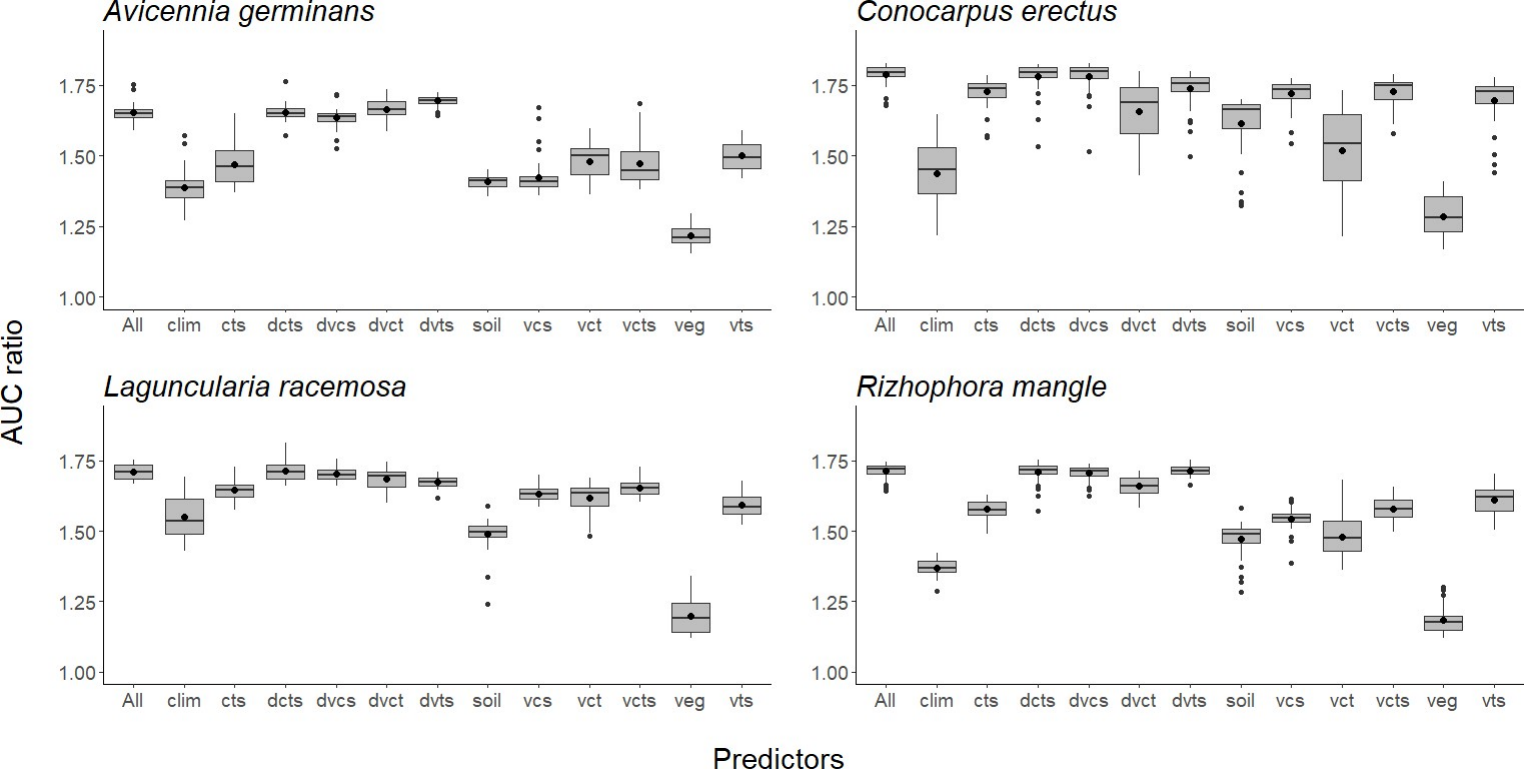
Comparison of AUC ratios between different sets of environmental layers for each of the four mangrove species: *Avicennia germinans*, *Conocarpus erectus*, *Laguncularia racemosa*, and *Rhizophora mangle*. The black bar inside the box represents the median, the black dot inside the box is the mean, edges of the box are the upper and lower quartile boundaries, and whiskers denote points within the 1.5 times the interquartile range of the upper limit. Atypical values of the distribution are indicated with black dots. Each letter is a class of predictors: d = distance to coast, c = climate variables, s = soil variables, t = topography (digital terrain model), v = vegetation variables.

**Fig 4:**
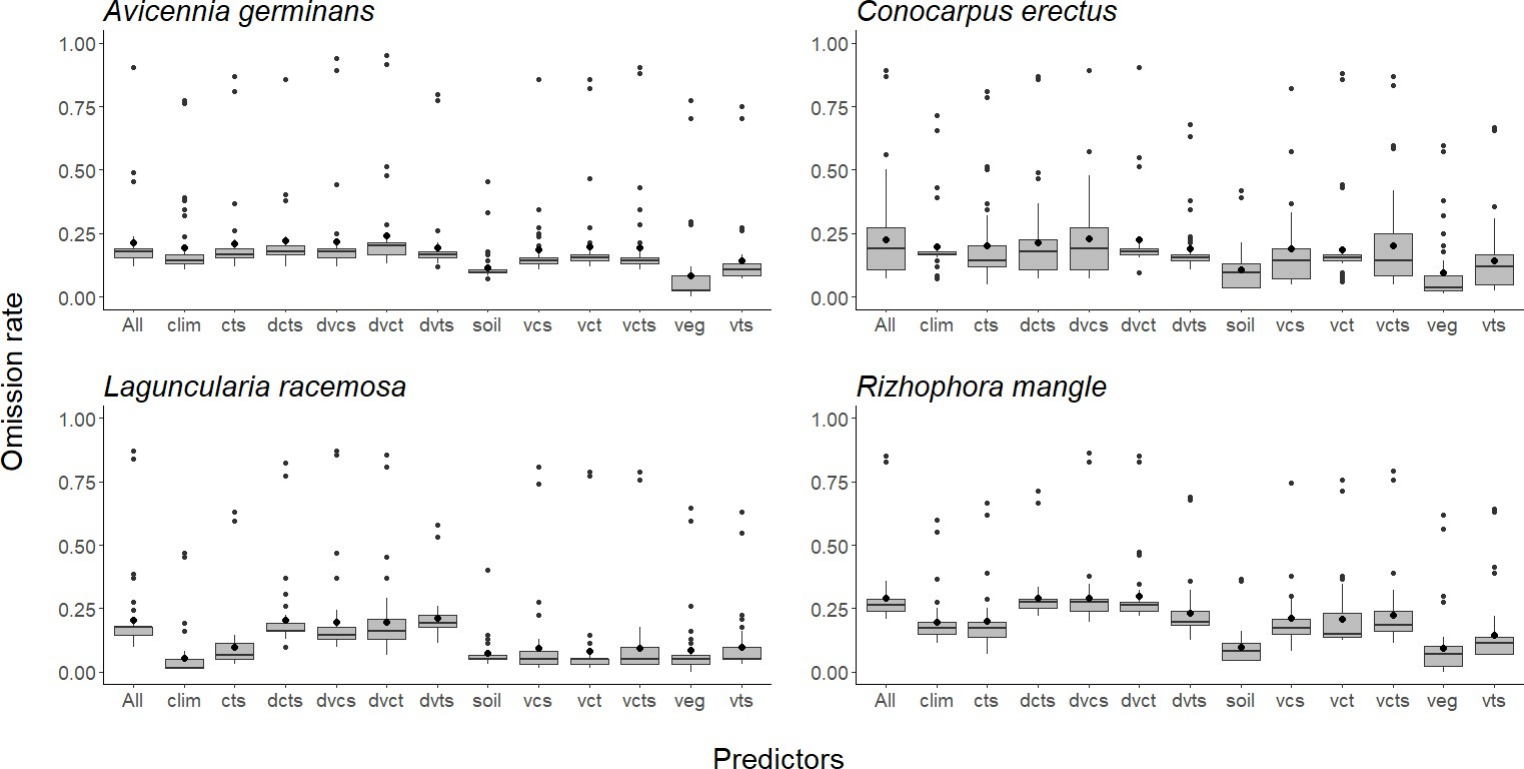
Comparison of omission rates between different sets of environmental layers for each of the four mangrove species: *Avicennia germinans*, *Conocarpus erectus*, *Laguncularia racemosa*, and *Rhizophora mangle*. The black bar inside the box represents the median, the black dot inside the box is the mean, edges of the box are the upper and lower quartile boundaries, and whiskers denote points within the 1.5 times the interquartile range of the upper limit. Atypical values of the distribution are indicated with black dots. Each letter is a class of predictors: d = distance to coast, c = climate variables, s = soil variables, t = topography (digital terrain model), v = vegetation variables.

**Fig 5:**
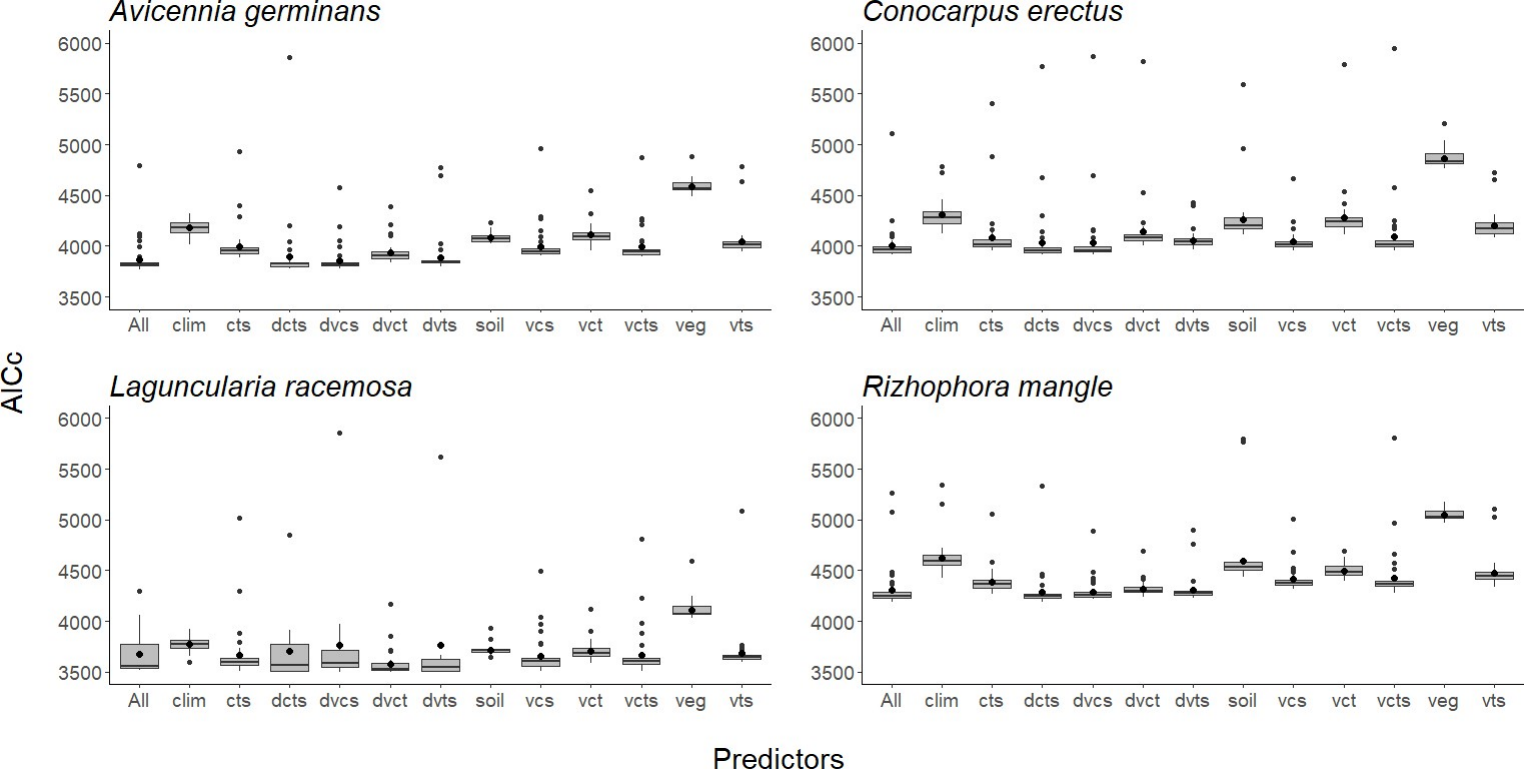
Comparison of Akaike information criterion values (AICc) between different sets of environmental layers for each of the four mangrove species: *Avicennia germinans*, *Conocarpus erectus*, *Laguncularia racemosa*, and *Rhizophora mangle*. The black bar inside the box represents the median, the black dot inside the box is the mean, edges of the box are the upper and lower quartile boundaries, and whiskers denote points within the 1.5 times the interquartile range of the upper limit. Atypical values of the distribution are indicated with black dots. Each letter is a class of predictors: d = distance to coast, c = climate variables, s = soil variables, t = topography (digital terrain model), v = vegetation variables.

In general, the most important variables in models of all species were distance to the coast, elevation, and minimum temperature of the coldest month. Another important variable was soil organic carbon, which was important to models for all species except *A*. *germinans*. For all species except *L*. *racemosa*, precipitation of driest quarter also was important (Table 1). The environmental data set with the least predictive capacity in all species was the one that included the vegetation layers only (Figs 3, 4 and 5).

**Fig 6:**
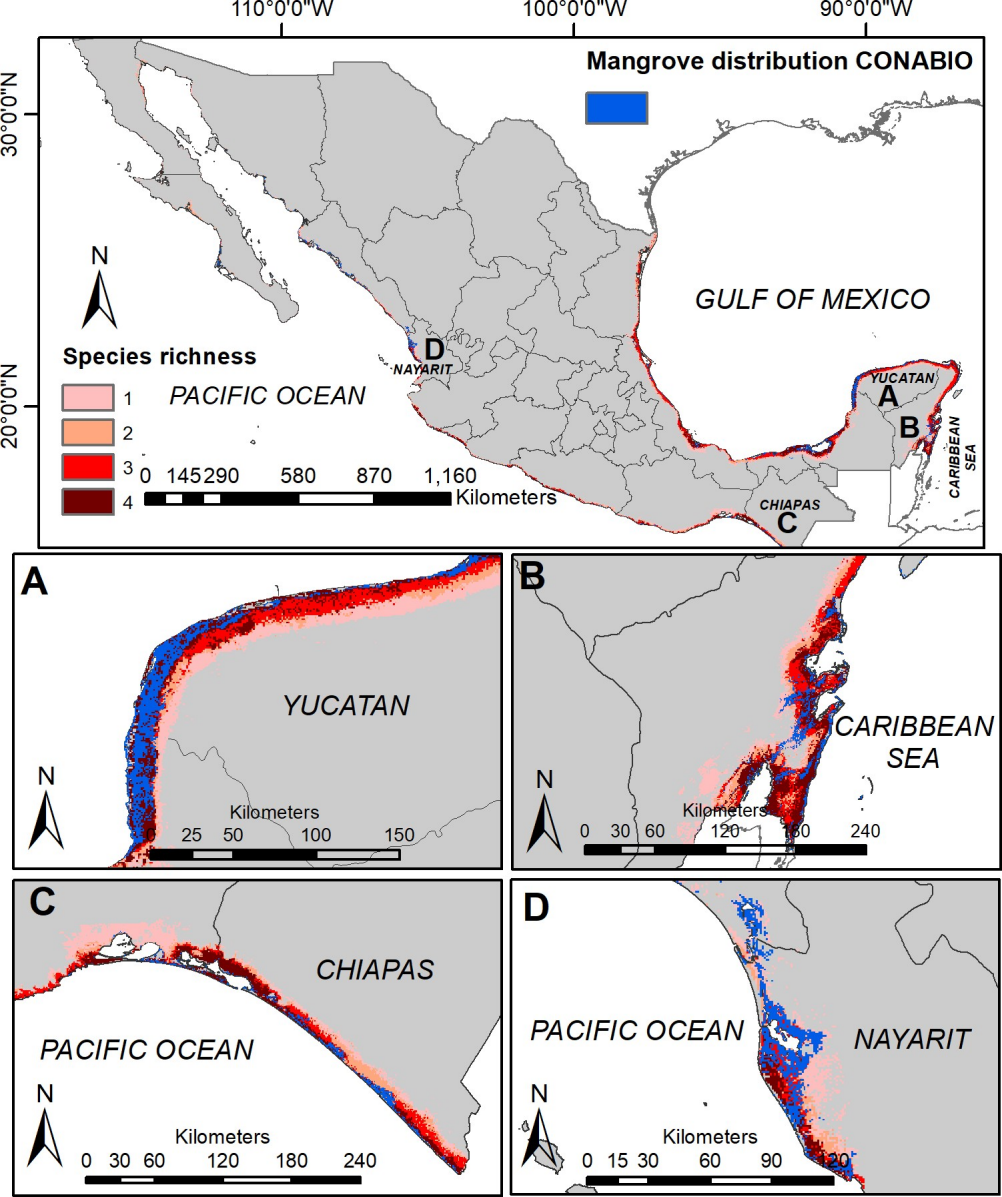
Predicted species richness of mangrove species across Mexico. Areas identified as suitable for all four species are shown in dark red, areas suitable for three species are in red, areas suitable for two species are in salmon, and areas suitable for only a single species are shown in pink. Richness patterns are shown for all of Mexico, (A) the northwestern coast of the Yucatan Peninsula, (B) the Mexican Caribbean, (C) southern Pacific Coast of Mexico, and (D) coastal Nayarit on the Pacific coast.

Our final, overall mangrove community model identified areas common to all species in parts of Veracruz, Campeche, Yucatán, Quintana Roo, and Chiapas, and also in small areas of Tamaulipas, Oaxaca, Guerrero, Colima, Jalisco, and Nayarit (Fig 6). Comparisons with the CONABIO map resulted in an omission error of 0.152, a commission error of 0.085, a correct classification rate of 0.913, and a TSS index of 0.762.

## Discussion

Eliminating older occurrence records and environmental outliers, potentially deriving from georeferencing errors or sink populations, allowed us to generate models that represented areas potentially occupied by mangrove species in Mexico at present [60]. However, even though eliminating clusters of records (spatial filtering) is generally advisable to reduce overfitting of models to regions that are sampled more intensively [60]. Our final models, constructed with spatially unfiltered records, estimated current mangrove distributions better. This outcome probably reflects the fact that the clusters in our data represent areas with greater abundance or mangrove coverage, thereby including relevant biological information about environmental suitability for mangrove species.

### Modeling of environmental suitability and potential distribution

Our models showed that the Mexican distributions of *R*. *mangle* and *A*. *germinans* are broader in comparison with those of the other two species, probably because they are the species with the broadest physiological tolerances, at least regarding temperature and salinity [61]. This tolerance range is also observed in the results of Record et al. [31], in which *A*. *germinans* is the species that best responds to low temperatures, and *R*. *mangle* the second most tolerant; the species most affected was *L*. *racemosa*. *Rhizophora mangle* is a species typical of coastlines, where it often forms masses in the intertidal zones of coastal lagoons and estuaries that are influenced by salt water. Its best development occurs at sites protected against effects of strong waves, and on shallow coastlines with little slope where the tide enters more easily [62].

The estimated distribution of *C*. *erectus* extended further inland, reflecting the fact that its distribution in natural environments is characteristically behind the other species of the mangrove community. This species is not considered to be a true mangrove (i.e., it does not have pneumatophores and is not viviparous; [63]), but rather is considered as a mangrove-associated species. *Conocarpus erectus* can tolerate high salinity and dry soils, but can also grow within or close to freshwater areas [63]. *Laguncularia racemosa* generally prospers on the shores of coastal lagoons, protected bays, and riverine estuaries, all areas that receive sea water inflow [61]. Overall, *L*. *racemosa* is less tolerant of high salinity and temperature levels than *R*. *mangle* and *A*. *germinans*, although it does tolerate flooding by brackish or fresh water [31, 63].

### Importance of variables

Dimensions of distance to coast, temperature, precipitation, and topography were consistently selected as best environmental predictors in shaping mangrove species’ distributions in Mexico, and these variables have proven to be important in mangrove models at broader scales [60].These variables are also known to influence the composition of mangrove communities, their phenological patterns, their productivity, and the distribution of this ecosystem under climate change scenarios [33, 43, 61, 64–66].

Here, sets of predictor variables that combined climate, topography and soil variables resulted better than sets consisting of climate, soil, or vegetation variables only. The latter dimension (vegetation) had inferior performance, possibly because the vegetation layers were values of the normalized difference vegetation index (NVDI). NDVI can be a proxy for more direct variables that influence the physiology of species at local rather than regional extents [44, 67]. The vegetation layers provided additional information when tested in combination with other variables, but were the worse predictors on their own.

In contrast, soil variables, particularly organic carbon, had strong contributions in all models. Indeed, soils are important components in mangrove development and distribution, as they serve as carbon sinks, sources of resources, and transformers of nutrients and other chemical contaminants [68]; hence, they impact water quality and productivity of the ecosystem. These soils are organic and hydromorphic [68], attributes that are probably reflected in the set of soil variables that we employed. This study is one of the few studies of mangrove species’ distributions in which soil variables have been incorporated, and resulted highly relevant to the models [69]. Across all predictor variable sets, the best models where those in which distance to coast was incorporated. This variable has been used widely in studies of diversity, composition and distribution of marine and coastal organisms [70–73]. This variable participated in the best models for at least three of the mangrove species. An important characteristic of mangroves is their coastal distribution, associated with the frequency and duration of tides [43, 74]. Tides influence factors such as salinity and flooding, which are key in restricting mangroves to coastal areas [75]. Distance to coast as an environmental variable likely functions as an indirect variable or a proxy of salinity and/or flooding [76], since it does not have a direct effect on the physiology of the mangrove species.

On the other hand, in the relatively few coastal areas of the country where mangroves are indeed present more than 20 km inland, models based on environmental predictors without the distance to coast layer were better, in that they represented accurately the inland mangrove patches reported by CONABIO [3], whereas models that included the distance to coast layer did not. These areas are few around the country, occurring only in south-central Quintana Roo and southern Campeche. As such, the distance to coast variable should be used with caution, as it may blind models to uncommon, but important, areas where mangroves are present further inland than is the norm.

In the set of climate variables, precipitation of the driest month and of the driest quarter of the year were important for *A*. *germinans* and *L*. *racemosa*, probably because rainfall is scarce during the driest months of the year, and salinity increases in the mangrove areas. This effect favors the presence of *A*. *germinans* but restricts that of *L*. *racemosa* owing to its low tolerance to salt [31, 63]. Temperature of the coldest month had an important contribution of in our models, and that coincides with the work of Cavanaugh et al. [61, 77] and Record et al. [31], who reported that low temperatures limit the distributions of mangrove species at higher latitudes.

### Richness and community map

The surface area that we estimated for mangroves in Mexico (18,456 km^2^) was more extensive than that represented in the map of CONABIO [3]. However, our models represent a geographic perspective on current favorable environmental conditions for the distribution of the species studied here. Differences in the extent of mangrove distribution between the Gulf of Mexico and the Pacific Ocean are consequence of distinctive climatic dynamics and geomorphological features. For instance, trade winds produce a circulation wind pattern ‘east-west’, which in turn makes the Gulf of Mexico coast wetter and warmer than the Pacific coast, favoring diversity and composition of mangroves in the former. In addition, geomorphology of the Gulf of Mexico coast is more suitable for mangrove establishment since estuaries and coastal lagoons provide them continental runoff water and protection from adverse weather conditions [35]. On the other hand, the narrower distribution of mangrove predicted in the north of the Gulf of Mexico in relation to the south coincides with Lot et al. [78] who mention that mangroves in this region are structurally simpler and less diverse because of the low temperatures and precipitation [78].

Several factors may nonetheless mean that the mangrove could be absent, in spite of the existence of adequate environmental conditions for their presence. A clear example is land use change: favorable conditions are manifested, but mangroves might be absent because of human-mediated clearing, or because of natural disturbances such as storms or hurricanes, which are particularly common in the Gulf of Mexico region and the Mexican Caribbean [3, 79]. Another reason may be that other environmental dimensions not considered here are modulating the local and regional distributions of mangrove species (i.e. intra- and inter-specific interactions, tidal dynamics, hydrology, accretion and coastal erosion).

## Conclusions

In view of the outcomes obtained in this project, we conclude that use SDM to obtain estimates of the potential areas of distribution of mangrove communities will provide reliable information even in complex regions like Mexico. Across broad areas, the major features of the distribution of this ecosystem are set by climate and topography. However, at local scales, models can be improved via incorporation of environmental layers that provide data more closely related to the species’ physiology, and regarding factors manifested at finer spatial scales.

This work therefore provides valuable information at regional scales and at the community level, as well as a complementary viewpoint from which to understand coarse-scale ecology of mangroves. Insights include identification of environmental predictors that correlate with each species’ geographic distribution and a potential species richness map that could be used as input to other biodiversity analysis (e.g., SESAM) [80] and other macroecological analyses. In addition, for other countries lacking a mangrove monitoring program such as the implemented by CONABIO in Mexico, SDMs could be the only effective way to study this ecosystem at national scale.

## Supporting information

S1 File (RAR)
